# Comparing neuromotor functions in 45- and 65-year-old adults with 18-year-old adolescents

**DOI:** 10.3389/fnhum.2023.1286393

**Published:** 2023-11-15

**Authors:** Tanja H. Kakebeeke, Aziz Chaouch, Jon Caflisch, Dominique A. Eichelberger, Flavia M. Wehrle, Oskar G. Jenni

**Affiliations:** ^1^Child Development Center, University Children’s Hospital Zurich, Zurich, Switzerland; ^2^Children’s Research Center, University Children’s Hospital Zurich, Zurich, Switzerland; ^3^Department of Epidemiology and Health Systems, Quantitative Research, Center for Primary Care and Public Health (Unisanté), University of Lausanne, Lausanne, Switzerland; ^4^Department of Neonatology and Intensive Care, University Children’s Hospital Zurich, Zurich, Switzerland; ^5^University of Zurich, Zurich, Switzerland

**Keywords:** aging, motor performance, associated movements, Zurich Neuromotor Assessment, last-in-first-out retrogenesis hypothesis

## Abstract

**Aim:**

This cross-sectional analysis investigates how neuromotor functions of two independent cohorts of approximately 45- and 65-year-old individuals are different from 18-year-old adolescents using the Zurich Neuromotor Assessment-2 (ZNA-2).

**Methods:**

A total of 186 individuals of the Zurich Longitudinal Studies (ZLS) born in the 1950s (mean age 65.1 years, SD = 1.2 year, range of ages 59.0–67.5 years, *n* = 151, 82 males) and 1970s (mean age 43.6 years, SD = 1.3 year, range of ages 40.8–46.6 years, *n* = 35, 16 males) were tested with the ZNA-2 on 14 motor tasks combined in 5 motor components: fine motor, pure motor, balance, gross motor, and associated movements. Motor performance measures were converted into standard deviation scores (SDSs) using the normative data for 18-year-old individuals as reference.

**Results:**

The motor performance of the 45-year-old individuals was remarkably similar to that of the 18-year-olds (SDS from −0.22 to 0.25) apart from associated movements (−0.49 SDS). The 65-year-olds showed lower performance than the 18-year-olds in all components of the ZNA-2, with the smallest difference observed for associated movements (−0.67 SDS) and the largest for gross motor skills (−2.29 SDS). Higher body mass index (BMI) was associated with better performance on gross motor skills for 45-year-olds but with worse performance for 65-year-olds. More educational years had positive effects on gross motor skills for both ages.

**Interpretation:**

With the exception of associated movements, neuromotor functions as measured with the ZNA-2 are very similar in 45- and 18-year-olds. In contrast, at age 65 years, all neuromotor components show significantly lower function than the norm population at 18 years. Some evidence was found for the last-in-first-out hypothesis: the functions that developed later during adolescence, associated movements and gross motor skills, were the most vulnerable to age-related decline.

## Introduction

Neuromuscular function, by which the muscles are controlled by the nervous system, changes dramatically over the human lifespan (Haywood and Getchell, 2014). Across childhood and adolescence, rapid increases in function take place due to growth and maturation of the neuromuscular system, and later in life more gradual decreases occur due to aging (Vandervoort, 2002; Maes et al., 2017). The stage of young adulthood in the 20s and early 30s displays the highest neuromuscular functions (Vandervoort, 2002; Haywood and Getchell, 2014; JafariNasabian et al., 2017). The subsequent loss in neuromuscular function and the capacity to maintain a healthy, active lifestyle can be attributed to the progressive decline of skeletal muscle mass with aging (Metter et al., 2002, 2005; Koopman and van Loon, 2009; Power et al., 2013). In young adults, lean muscle mass contributes up to about 50% of total body weight, but this declines to 25% by 75–80 years of age (Koopman and van Loon, 2009). It is for this reason that reductions in gait speed are strongly correlated with age (Stanaway et al., 2011; Rasmussen et al., 2019). Additionally, there is a disproportionate decrease of motoneurons in the spine, especially of those motoneurons belonging to the fast muscle fibers, which manifests itself as a reduction in strength and coordination over age (Hepple and Rice, 2016) and an increase in gait variability (Pieruccini-Faria et al., 2021).

There is evidence that muscle structure and neuromuscular function are associated with brain structure (Kilgour et al., 2014); nowadays, structural changes in the human brain across the lifespan can be made visible with MRI, enabling this association to be studied in depth (Douaud et al., 2014; Slater et al., 2019; Kiely et al., 2022). Such studies have led to the hypothesis that brain maturation in early life is mirrored during aging later in life (Douaud et al., 2014). Thus, Slater et al. (2019) postulated that the structures that develop ontogenetically most slowly are the most vulnerable to age-related decline; they called this the last-in-first-out retrogenesis hypothesis.

However, how neuromaturation early in life and later aging processes in the brain are reflected in neuromotor performance over the life span is not known (Power et al., 2013; Haywood and Getchell, 2014). Various motor tests have been used to demonstrate an age-related improvement in diverse neuromotor functions from childhood to young adulthood (Largo et al., 2001a,b; Bruininks and Bruininks, 2005; Henderson et al., 2007; Kakebeeke et al., 2018). Furthermore, a deterioration in neuromotor functions is observed with age in elderly individuals (Power et al., 2013; Haywood and Getchell, 2014; JafariNasabian et al., 2017). For example, balance is known to be a function that deteriorates strongly with age (Springer et al., 2007), and the function of bimanual tasks is negatively correlated with age (Maes et al., 2017). However, these declines in performance are less well described than the improvement in neuromotor function during early development.

A comprehensive neuromotor assessment including young and old individuals, covering various neuromotor functions and differentiating fine and gross motor performance would provide insights into functional changes across the lifespan (Kakebeeke et al., 2018). As far as the neuromotor assessment for young individuals is concerned, the norm data of the 18-year-olds are already described elsewhere (Kakebeeke et al., 2018); data of adult ages are lacking, however. Such an approach into adulthood can inform researchers and practitioners about which functions stay constant over time and which are prone to deterioration.

Working from the last-in-first-out retrogenesis hypothesis, we addressed whether some neuromotor functions deteriorate earlier than others and if so, whether these functions are also those that are attained the latest during adolescence. Thus, we assessed one group of middle-aged (~45 years) individuals and one group of young elderly (~65 years) individuals and compared their data with that of a norm population of adolescents (18 years) tested with the Zurich Neuromotor Assessment-2 (ZNA-2) (Kakebeeke et al., 2019).

## Methods

### Participants and study procedure

Data for the current analyses on neuromotor performance are derived from the most recent assessments of the Zurich Longitudinal Studies (ZLS), including the participants now approximately 45 and 65 years old enrolled from the greater Zurich area in Switzerland. As part of the comprehensive assessment, participants performed the ZNA-2, were measured for height and weight, and completed a questionnaire on sociodemographics. For a more detailed description of the study procedures, we refer to the study protocol by Wehrle et al. (2021). The first group, part of the ZLS-1, included participants aged 59.0–67.5 years (mean age 65.1, SD = 1.2 year, *n* = 151, 82 males). The second group, part of the ZLS-2, comprised adults (between 40.8 and 46.7 year old) (mean age 43.6 years, SD = 1.3 year, range of ages 40.8–46.6 years, *n* = 35, 16 males). Participants from the two groups were tested by the same examiners, who were trained and supervised by the same expert (THK) that supervised, gathered and published the original data for the ZNA-2 normative sample (Kakebeeke et al., 2018), thereby minimizing changes in test modus. The study was approved by the ethical committee of the Canton of Zurich, Basec-Nr. 2018–00686.

### Test battery

#### Zurich Neuromotor Assessment-2 (ZNA-2)

The ZNA-2 is a standardized procedure for assessing the speed of several neuromotor tasks with timed performance and the quality of movements: the intensity of associated movements occurring on the contralateral side not contributing to the task. Fine and gross motor performance are differentiated into five components: fine motor (FM), pure motor (PM), balance (BA), gross motor (GM), and contralateral associated movements (CAMs) (Kakebeeke et al., 2018, 2019). See Table 1 for an overview of the tasks. The ZNA-2 assesses the variability and evolution of neuromotor performance as a function of age and sex (Largo et al., 2001a,b; Kakebeeke et al., 2018, 2019). The data gathered with this study on the ~45- and ~65-year-olds are compared with the data published elsewhere (Kakebeeke et al., 2018).

**Table 1:** Overview of the tasks of the ZNA-2 (Kakebeeke et al., 2019).

Motor components	Tasks
Fine motor (FM)	Pegboard (+CAMs)		Bolts (+CAMs)		Beads		Pure motor (PM)	Repetitive movements	Fingers
Hand								
Foot								
Alternating movements	Hand (pro/supination) (+CAMs)
	Foot (heel–toe alternation) (+CAMs)
	Sequential movements Fingers (+CAMs)
Balance (BA)	Standing on one leg (eyes open)	
	Standing on one leg (eyes closed)	
Gross motor (GM)	Jumping sideways	
	Chair-rise	
	Standing long jump	

#### Body mass index (BMI)

Height was measured with a stadiometer to the nearest 0.1 cm and weight to the nearest 0.1 kg (Seca, Basel, Switzerland). BMI was calculated from these measures (kg/m^2^).

#### Socio-economic status (SES)

As some of the ~65-year-olds had already retired at the time of testing, we used the highest education as a proxy for SES rather than occupation. The highest education achieved was categorized according to the Swiss Household Panel (Voorpostel et al., 2016) on a scale ranging from 0, incomplete compulsory school, to 16, having completed a PhD.

### Statistical analysis

Motor performance and intensity of CAMs of the ~45-year-olds and ~65-year-olds were converted into standard deviation scores (SDSs) for the five components of the ZNA-2 (Kakebeeke et al., 2018). An SDS is a gender-adjusted standardized measure of motor performance that is approximately normally distributed in the reference population (here 18-year-olds), with a mean of zero and a standard deviation of one. Positive SDSs refer to above-average performance, and negative SDSs refer to below-average performance. A linear regression model (analysis of variance) was then used to model SDSs as a function of the age group while controlling for gender differences. This additive model assumes that the age effect is the same in males and females. Additionally, a model allowing different age effects in males and females by including interaction terms was also fitted, with corresponding results presented in the Appendix. The modelling was performed separately for FM, PM, BA, GM, and CAMs. This approach allowed the estimation of differences in average SDSs between each cohort and the reference population as well as between the two cohorts themselves. Next, we used an analysis of covariance to quantify the linear effect of BMI and SES on motor SDS within each age group separately while controlling for gender and age variability within a given age group. Within each age group, the association between BMI and SES was also estimated using Spearman’s rank correlation coefficient. All statistical analyses were conducted with R version 4.2.2 (R Core Team, 2022) using a level of statistical significance set at 5%.

## Results

**Figure 1:**
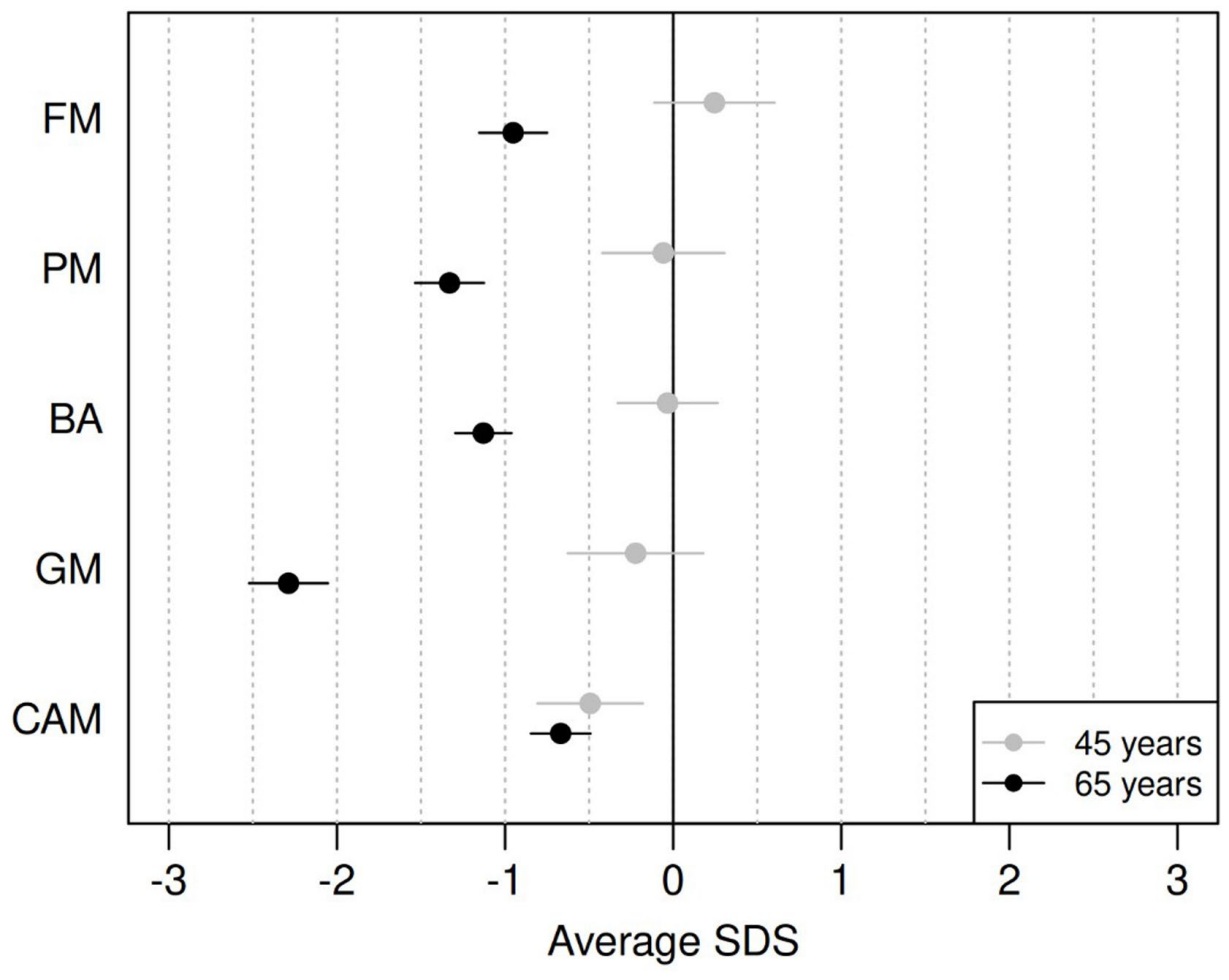
Differences between average SDS on ZNA-2 components for the ~65-year-olds (black) and for the ~45-year-olds (grey), compared to the norm population (18-year-olds). FM, fine motor; PM, pure motor; BA, balance; GM, gross motor; CAMs, contralateral associated movements. Horizontal bars refer to 95% confidence intervals for the mean SDS value.

Table 2 reports the average SDSs for the five components for the two age groups and the estimated difference in average SDS between the two cohorts. A graphical representation of the results is provided in Figure 1. For the PM and FM components, the data from all participants was used; for the BA and GM components, some participants were unable to perform the tasks for various reasons, such as pain in the lower joints and vertigo. Data were thus missing from BA eyes open (*n* = 2), BA eyes closed (*n* = 3), jumping sideways (*n* = 19), chair rising (*n* = 5) and standing long jump (*n* = 15). All missing data except one for BA with eyes closed came from the group of ~65-year-olds.

**Table 2:** Average SDS with 95% confidence intervals (CI) for ZNA-2 components in each age group.

	Average SDS (95% CI) in comparison to the 18-year-olds		
Component	~45 years	~65 years	Difference
FM	0.25 (−0.11; 0.60)	**−0.95 (−1.15; −0.75)**	**−1.20 (−1.56; −0.84)**
PM	−0.06 (−0.42; 0.30)	**−1.33 (−1.54; −1.13)**	**−1.27 (−1.64; −0.91)**
BA	−0.03 (−0.33; 0.26)	**−1.13 (−1.30; −0.96)**	**−1.10 (−1.40; −0.80)**
GM	−0.22 (−0.63; 0.18)	**−2.29 (−2.52; −2.05)**	**−2.06 (−2.47; −1.66)**
CAMs	**−0.49 (−0.81; −0.18)**	**−0.67 (−0.85; −0.49)**	−0.18 (−0.49; 0.14)

Statistically significant results appear in bold. FM, fine motor; PM, pure motor; BA, balance; GM, gross motor; CAMs, contralateral associated movements.

The motor performance of the ~45-year-olds remained remarkably similar to that of the reference population of 18-year-olds, with average SDS ranging from −0.22 (GM) to 0.25 (FM). However, the ~45-year-olds showed significantly more CAMs with lower SDSs than the reference population (−0.49 SDS). The older cohort had significantly lower motor performance than the reference population across all components of the ZNA-2, with the largest difference seen in GM (−2.29 SDS).

The average BA performance with eyes open or closed did not differ between ~45-year-olds and 18-year-olds (0.05 SDS, 95% CI [−0.32; 0.43] and − 0.09 SDS, 95% CI [−0.34; 0.15], respectively). At ~65 years, BA was significantly lower for both tasks. The difference between the ~65-year-olds and the ~45-year-olds with eyes open was −0.71 SDS (95% CI [−1.09; −0.33]), whereas it was −1.26 SDS with eyes closed (95% CI [−1.51; −1.01]).

The performance in GM was more than 2 SDS lower in ~65-year-olds than in 18-year-olds. We separately studied the three tasks forming this component: jumping sideways, chair rising, and standing long jump. Among the ~65-year-olds, the poorest performance was observed in standing long jump (−2.42 SDS, 95% CI [−2.61; −2.23]), followed by jumping sideways (−1.62 SDS, 95% CI [−1.82; −1.41]), and chair rising (−1.06 SDS, 95% CI [−1.28; −0.83]), all statistically different from the reference values from 18-year-olds. For further details, see Appendix 1 on the task differences over the groups.

The results of the sex-adjusted analysis are presented in Appendix 2. In the ~45-year-olds, there were no significant differences between the sexes. In the ~65-year-olds, females did not differ in performances from males in FM, PM and in GM. However, females scored lower than males in BA (females: −1.38, males: −1.09 SDS) and CAMs (females: −1.03, males: -0.66 SDS), with statistically significant differences.

The mean BMI in ~45- and ~65-year-olds was 24.0 and 25.9, respectively (*p* = 0.011). The two cohorts had different distributions in SES, with a median of 14 (equivalent to finishing a bachelor’s degree) for the ~45-year-olds and 8.5 (equivalent to finishing teacher training course) (“Lehrerseminar”) or completing the general qualification for university entrance (“Matur”) for the ~65-year-olds (*p* < 0.001, Mann–Whitney *U*-test). The association between BMI and SES was negative in the two age groups, with rank correlation coefficients of −0.32 (*p* = 0.063) and − 0.09 (*p* = 0.268) at ~45 years and ~65 years, respectively, indicating that a lower BMI was associated with a higher SES. Consequently, we analyzed the effect of BMI and SES separately in each age group. Table 3 reports the changes in SDS associated with increases of 5 points on BMI and 6 points on SES in each age group after controlling for sex and age within each cohort. Such changes in BMI and SES correspond approximately to the interquartile range of either variable. An increase in BMI in the ~65-year-olds was associated with lower motor performance across all components of the ZNA-2. However, statistically significant effects were only observed in gross motor tasks. Participants in the upper quartile of BMI scored on average − 0.50 SDS and − 0.28 SDS lower than peers in the lower quartile for GM and BA, respectively. The same contrast in BMI resulted in better performance in these gross motor tasks among ~45-year-olds, with the difference in GM (0.59 SDS) being statistically significant. In general, BMI had a weak effect on motor performance in FM, PM, and CAMs in both age groups.

**Table 3:** Difference in motor SDS with corresponding 95% confidence intervals between participants at the upper quartile and those at the lower quartile of BMI or SES distribution, after controlling for sex and age variability within each age group separately.

	Effect of increasing BMI by 5 points (for fixed age, sex and SES)		Effect of increasing SES by 6 points (for fixed age, sex and BMI values)	
	~45 years	~65 years	~45 years	~65 years
FM	0.03 [−0.47; 0.53]	−0.17 [−0.36; 0.03]	0.34 [−0.20; 0.88]	0.13 [−0.09; 0.35]
PM	−0.28 [−0.85; 0.29]	−0.06 [−0.27; 0.15]	0.30 [−0.31; 0.91]	−0.11 [−0.34; 0.13]
BA	0.20 [−0.09; 0.50]	**−0.28 [−0.45; −0.10]**	0.15 [−0.16; 0.47]	**0.20 [0.00; 0.40]**
GM	**0.59 [0.15; 1.03]**	**−0.50 [−0.73; −0.26]**	**1.00 [0.53; 1.47]**	**0.31 [0.07; 0.55]**

Changes of 5 points in BMI and 6 points in SES correspond approximately to the interquartile range within each age group. Statistically significant effects are reported in bold characters. FM, fine motor; PM, pure motor; BA, balance; GM, gross motor; CAMs, contralateral associated movements.

The effect of SES on motor performance was generally weak. The only 2 exceptions were found in GM among the ~45-year-olds, with participants in the upper quartile of the SES distribution scoring on average 1.00 SDS above those in the lower quartile. A positive effect of SES on GM performance could also be found among the elderly but with a lower magnitude of 0.31 SDS. Similar effects of SES could be found for BA among the elderly.

## Discussion

In this cross-sectional study, the neuromotor functioning of two cohorts, one of ~45-year-olds and one of ~65-year-olds, was investigated and compared with the norm data of the ZNA-2 at 18 years (Kakebeeke et al., 2018). In general, the timed performance of the ~45-year-olds was comparable to that of 18-year-olds on fine motor (FM), pure motor (PM), balance (BA) and gross motor (GM) tasks. However, the ~45-year-olds had significantly higher intensities of contralateral associated movements (CAMs) than the reference population of 18-year-olds, resulting in negative average SDS. In contrast, all neuromotor functions were significantly lower for the ~65-year-old cohort, not only in comparison to the norm population but also compared to the ~45-year-olds. The smallest difference between the two cohorts was observed in CAMs (−0.18 SDS, not significant) and the largest was observed in GM (−2.06 SDS).

### Age differences

Our findings of lower performance in FM and PM in older adulthood compared to young adulthood are in line with Smith et al.’s results on fine motor hand movements during aging (Smith et al., 1999). These authors describe how the time required to perform fine motor tasks increases linearly with age. However, for particularly difficult motor tasks, the authors identified a non-linear relationship between motor performance and age, with the time required to complete the task increasing much faster among participants older than 60 years than among younger participants (Smith et al., 1999). This faster drop in performance after age 60 is accredited to a decreased activity of the neuronal cells responsible for the synthesis of dopamine, as shown in aging participants (McGeer et al., 1977). The dopaminergic nigrostriatal tract, originating in the basal ganglia, is important for the control of visually guided reaching and movements (McGeer et al., 1977). The tasks of the ZNA-2 that assess FM performance involve a strong visuomotor aspect, implying that the basal ganglia have to be active and any deterioration in these ganglia will impact the execution of more complex movements. Here, we only observed a deterioration in the PM and FM components in the ~65-year-olds and not in the ~45-year-olds, strongly supporting Smith et al.’s (1999) findings on increased performance times and McGeer et al. (1977) on the contribution of the basal ganglia to more difficult tasks.

We observed an increase in the intensity of CAMs at ~45 years, with no further significant deterioration by ~65 years. Indeed, a recurrence of CAMs has previously been described to take place across age (Baliz et al., 2005; Addamo et al., 2009; Koerte et al., 2010). CAMs are unintended movements of the distal parts of the extremities by the homologous muscles contralateral to the active site (Largo et al., 2001b; Cincotta and Ziemann, 2008; Koerte et al., 2010). Interestingly, in children and adolescents, there is a strong decrease in CAMs until about 10 years, after which there is a slower decrease until adolescence (Cincotta and Ziemann, 2008). This disappearance of CAMs coincides with the completeness in myelination of the callosal fibers, which are critical for information transfer (Mayston et al., 1999; Williams et al., 1999; Carlson, 2005; Cincotta and Ziemann, 2008; Tsvetanov et al., 2018; Bethlehem et al., 2022). The changes in CAMs in the ~45- and ~ 65-year-olds in comparison to the norm population align with studies on changes in white matter in the brain over the lifespan (Cincotta and Ziemann, 2008; Bartzokis et al., 2010; Bender et al., 2016; Slater et al., 2019; Bethlehem et al., 2022). We know that myelin content and axonal density decrease in the course of aging, leading to lower conduction velocities and less protection of the nerve fibers in older adults (Slater et al., 2019; Bouhrara et al., 2021). These changes in the structure of myelin are reflected in functional changes, as was shown by Gooijers and Swinnen (2014). According to their study, the function of the corpus callosum is determined by its degree of early maturation and deterioration at older age. Two roles are attributed to the corpus callosum: an excitatory role and an inhibitory one. The excitatory role consists mainly of the exchange and integration of information, and the inhibitory role allows functional specialization and independent processing of both hemispheres (Takeuchi et al., 2012; Gooijers and Swinnen, 2014). Thus, the inhibitory role of the corpus callosum is visible in decreased CAMs and in differential two-handed tasks such as in stringing beads, for which inhibition from one side to the other is necessary (Addamo et al., 2007; Cincotta and Ziemann, 2008). In adults, according to Cazeba’s (2002) model, which was elaborated by Baliz et al. (2005), increased age-related bilateral activation is associated with task performance. For exactly the same task, there is more activation and/or less inhibition in the corpus callosum with increasing age (Baliz et al., 2005). In fact, reduced performance in complex bimanual tasks occurs at advanced age, which is attributed to changes in the microstructure of the corpus callosum (Lebel et al., 2012; Leversen et al., 2012; Gooijers and Swinnen, 2014).

Our findings of the reappearance of CAMs by mid-adulthood alongside our previous findings that CAMs disappear latest in adolescence (Kakebeeke et al., 2018) mirrors the inverted U shaped curve relationship between white matter volume and age described by Leversen et al. (2012). They also support the last-in-first-out retrogenesis hypothesis: the CAMs are the first to recur with older age, in the current study with ~45 years, as they were ontogenetically the slowest to disappear (Douaud et al., 2014; Slater et al., 2019). Our study, thus, confirms the idea of a close relationship between the functional phenomenon of CAMs and structural changes in the brain.

At ~45 years the difference in BA from the norm population was not significant. At ~65 years, a difference in performance was found for BA between the ~45- and ~ 65-year-old groups. At ~65 years, the results for BA changed significantly for both tasks, eyes open and closed. However, the difference between the ~65- and the ~45-year-olds was much less with eyes open (−0.70 SDS) than with eyes closed (−1.24 SDS). This suggests that performance on tasks without visual guidance deteriorates more rapidly with age than performance on tasks that need visual guidance. These results are supported by Springer et al. (2007), who published normative values of the one leg stance test with eyes open and closed. In this study, participants were between 19 to 99 years old. After age 60, the decline in values for standing on one leg with eyes open became significantly different from the youngest (norm) age group from 18 to 39. However, the decline in one leg balance with the eyes closed in comparison to the norm group began 10 years earlier, because this is a much more complex task. Interestingly, standing on one leg with the eyes closed is also a very difficult task for children below 6 years, whereas at 18 years, the majority of the adolescents were able to perform this task (Kakebeeke et al., 2018). Again, the findings of the current study support the last-in-first-out-retrogenesis hypothesis of aging (Slater et al., 2019) on the basis that the function of standing on one leg with eyes closed was the latest to appear during maturation and the earliest function to deteriorate in older adults.

The GM component showed a large difference between ~45- and ~ 65-year-olds. In our assessment, the GM component is measured with three exercises evaluating endurance in the lower extremities mostly for sideward jumping, force mostly for chair rising, and explosive force with a standing long jump. Muscular function declines generally over age due to the loss of fibers, which occurs among fast and slow twitch fibers (Deschenes, 2004; Arking, 2006; Koopman and van Loon, 2009; JafariNasabian et al., 2017). However, disproportionate changes occur in maximal explosive force over age due to a stronger decline in the cross-sectional area of the fast muscle fibers over age (Deschenes, 2004; Samuel et al., 2012; Hepple and Rice, 2016). Thus, for the task standing long jump, the older participants showed a deterioration of −2.42 SDS in comparison to the norm data, whereas the deterioration of general force was much less: for sideward jumping, −1.62 SDS, and for chair rising, −1.06 SDS. This suggests that the strong deterioration in GM is mainly driven by the poor performance of the standing long jump in older participants. Interestingly, standing long jump performance, which takes a long time to develop, with males not reaching their full potential even at ~18 years old (Kakebeeke et al., 2018), is also the first ability to deteriorate in adults. Again, the findings of the current study confirm the last-in-first-out retrogenesis hypothesis at the level of neuromotor function.

### Sex differences

In ~45-year-old individuals, no sex difference was found in any of the five components. At ~65 years, males and females had both deteriorated in their performance, although the females had significantly lower scores than males in BA and CAMs. Because at age 18 years the females were earlier than males in achieving optimal scores in BA and CAMs (Kakebeeke et al., 2018), in line with the last-in-first-out retrogenesis hypothesis, at ~65 years, the females may also be earlier in their decline.

### Body mass index (BMI) and socioeconomic status (SES) as confounding variables

A higher BMI at ~45 years had a positive impact on the GM component, indicating that at this age the effect of having more weight influenced antigravity skills positively, which is not what we expected. As the BMI in this age group was not significantly linked to the standing long jump, requiring explosive muscle force, the positive relationship might have been caused by the tasks that require coordinatively less difficult pure muscle force, such as chair rising and jumping sideways. It should be noted that BMI is not the best proxy for the distribution of fat and lean muscle mass in the human body because the density of fat is much lower (Bozoyan and Wolbring, 2011). However, it is the most easily applicable parameter for weight. For this reason, sporty people with little fat but very much muscle mass are bound to have a high BMI. This could explain the unexpected result that a higher BMI in ~45-year-olds is associated with better GM. In contrast, at ~65 years, a physiological decrease in muscle mass and increase in body fat has taken place (Koopman and van Loon, 2009), so here the negative impact of BMI on BA and GM becomes obvious. This effect is even aggravated by the general increase in BMI between the 45- and 65-year-olds. BA and GM are both dependent on gravity, like the standing long jump, so it is not surprising that the estimate for this task was negative (−0.37).

SES was positively related to GM and the standing long jump at ~45 years. At ~65 years, a positive association was also found with BA, GM, and the standing long jump. This finding indicated that the more educated people were, the better were their gross motor skills. It has previously been shown that an active, healthy lifestyle is more commonly followed by more educated people (Kantomaa et al., 2016; Nikitara et al., 2021). This likely explains the associations that were found in the current study.

### Strength and limitations

This study provides insight into potential age-related changes in the human neuromuscular system (Yakovlev and Lecours, 1967; Mattay et al., 2002; Lebel et al., 2012; Kilgour et al., 2014; Slater et al., 2019; Buyanova and Arsalidou, 2021) by using a performance-based task battery to assess neuromotor functioning. This shows that a relative simple motor test such as the ZNA-2 is sufficiently sensitive to provide information about the underlying neurological structures.

One of the limitations of this study is that we only assessed neuromotor function at two ages and compared them with a reference population at age 18 years. This cross-sectional approach does not enable us to describe true developmental changes over age. Furthermore, in the study of the now ~45-year-olds, fewer individuals were enrolled than in that of the ~65-year-olds; therefore, the group sizes were unbalanced. Another limitation of the present study is that some ~65-year-olds were unable to perform GM and to a lesser extent BA tasks due to pain or arthrosis in the hip, knee, or ankle joints, resulting in missing data. Unsurprisingly, the older the participants become, the higher the chances of physical restrictions or pain in joints of the lower limbs which is common in Switzerland. However, such missing data are not missing at random because they arise from the inability to perform the task. In this study, we performed a complete-case analysis. Consequently, we note that the average GM performance in the ~65-year-old group reported in Table 2 likely overestimates the true GM performance of ~65-year-olds in the population. We would thus anticipate an even larger drop in GM performance between ~45 years and ~65 years. A further limitation of this study is that the participants’ BMI was measured as an indicator of their weight status. For this reason, any difference between the two groups in fat-free mass must remain a subject of speculation. Probably, the ~45-year-olds had more fat-free mass than the ~65-year-olds.

## Conclusion

The current study demonstrates that some neuromotor functions are more vulnerable than others to age-related decline. This provides some evidence for the last-in-first-out hypothesis: the functions that developed later during adolescence, associated movements and gross motor skills, were the most vulnerable to age-related decline. The neuroanatomical and neurophysiological correlates of the nervous system may be closely reflected in changes in human neuromotor function. Testing motor performance with a stopwatch and tape measure is easily applicable in clinical practice and an inexpensive way to gain insight into changes in the brain over the lifespan.

## Data availability statement

The raw data supporting the conclusions of this article will be made available by the authors, without undue reservation.

## Ethics statement

The studies involving humans were approved by Ethical committee of the Canton of Zurich, Basec-Nr. 2018–00686. The studies were conducted in accordance with the local legislation and institutional requirements. The participants provided their written informed consent to participate in this study.

## Author contributions

TK: Conceptualization, Data curation, Funding acquisition, Writing – original draft, Writing – review & editing. AC: Data curation, Formal analysis, Writing – review & editing. JC: Conceptualization, Validation, Writing – review & editing. DE: Data curation, Validation, Writing – review & editing. FW: Funding acquisition, Supervision, Writing – review & editing. OJ: Conceptualization, Funding acquisition, Resources, Supervision, Writing – review & editing.
