# Supplementary material for: Comparing neuromotor functions in 45- and 65-year-old adults with 18-year-old adolescents
Source: Front Hum Neurosci. 2023 Nov 15;17:1286393. doi: 10.3389/fnhum.2023.1286393 (PMC10684742; doi:10.3389/fnhum.2023.1286393)

Appendix 1:

Change in standard deviation scores over the tasks at ~45 and at ~65 years of age.
Abbreviations: FM: fine motor; PGB: pegboard; BLT: bolts; BDS: beads;
PM: pure motor; RFT: repetitive foot movements; RHD: repetitive hand movements; RFG: repetitive finger movements; AFT: alternating foot movements; AHD: alternating hand movements; SFG: sequential finger movements; BA: balance; BAO: balance, eyes open; BAC: balance, eyes closed; GM: gross motor; JSW: jumping sideways; CHR: chair rising; SLJ: standing long jump.

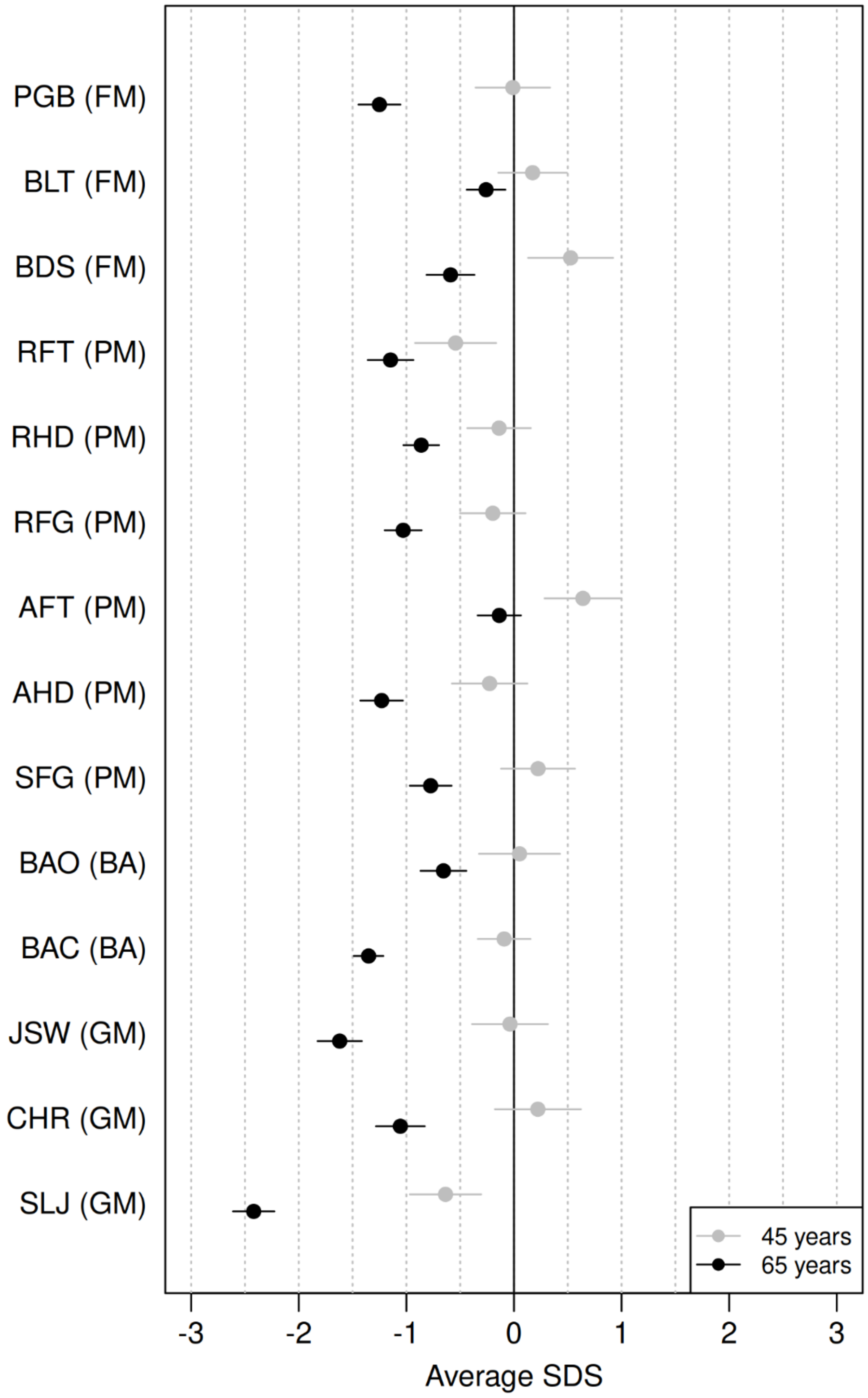

Supplement: Supplementary file 1 [file Table_1.pdf]
