# Supplementary material for: Comparing neuromotor functions in 45- and 65-year-old adults with 18-year-old adolescents
Source: Front Hum Neurosci. 2023 Nov 15;17:1286393. doi: 10.3389/fnhum.2023.1286393 (PMC10684742; doi:10.3389/fnhum.2023.1286393)
Supplement: Supplementary file 2 [file Table_2.pdf]

Appendix 2:

Average SDS with 95% confidence intervals (CI) for ZNA-2 components in each age group and sex. Statistically significant results are outlined in bold characters. Abbreviations: FM: fine motor; PM: pure motor; BA: balance; GM: gross motor; *p-value for sex difference.

		Average SDS (95% CI)		
Component	Age group	Males	Females	p-value*
FM	~45 years	**0.52 (0.05; 1.00)**	-0.11 (-0.55; 0.32)	0.053
	~65 years	**-1.01 (-1.22; -0.80)**	**-1.01 (-1.24; -0.79)**	0.961
	Diff.	**-1.53 (-2.05; -1.01)**	**-0.90 (-1.39; -0.41)**	0.084
PM	~45 years	-0.24 (-0.72; 0.25)	0.15 (-0.30; 0.59)	0.252
	~65 years	**-1.30 (-1.51; -1.08)**	**-1.31 (-1.55; -1.08)**	0.906
	Diff.	**-1.06 (-1.59; -0.53)**	**-1.46 (-1.97; -0.96)**	0.278
BA	~45 years	-0.26 (-0.65; 0.14)	-0.04 (-0.41; 0.33)	0.421
	~65 years	**-1.09 (-1.26; -0.91)**	**-1.38 (-1.58; -1.19)**	**0.024**
	Diff.	**-0.83 (-1.26; -0.40)**	**-1.35 (-1.77; -0.93)**	0.088
GM	~45 years	**-0.54 (-1.07; -0.01)**	0.09 (-0.40; 0.58)	0.086
	~65 years	**-2.22 (-2.47; -1.98)**	**-2.32 (-2.60; -2.05)**	0.584
	Diff.	**-1.68 (-2.27; -1.10)**	**-2.42 (-2.98; -1.86)**	0.076
CAMs	~45 years	**-0.55 (-0.97; -0.13)**	**-0.78 (-1.17; -0.40)**	0.424
	~65 years	**-0.66 (-0.84; -0.47)**	**-1.03 (-1.23; -0.82)**	**0.009**
	Diff.	-0.10 (-0.56; 0.35)	-0.24 (-0.67; 0.19)	0.669
